# Influence of *Eugenia uniflora* Extract on Adhesion to Human Buccal Epithelial Cells, Biofilm Formation, and Cell Surface Hydrophobicity of *Candida* spp. from the Oral Cavity of Kidney Transplant Recipients

**DOI:** 10.3390/molecules23102418

**Published:** 2018-09-20

**Authors:** Luanda B. F. C. Souza, Walicyranison P. Silva-Rocha, Magda R. A. Ferreira, Luiz Alberto L. Soares, Terezinha I. E. Svidzinski, Eveline P. Milan, Regina H. Pires, Ana Marisa Fusco Almeida, Maria José S. Mendes-Giannini, Guilherme Maranhão Chaves

**Affiliations:** 1 Departamento de Análises Clínicas e Toxicológicas, Universidade Federal do Rio Grande do Norte-UFRN, Rua Gal, Gustavo Cordeiro de Faria, S/N, Petrópolis, Natal-RN 59084-100, Brazil; luanda_canario@hotmail.com (L.B.F.C.S.); wrplinio@hotmail.com (W.P.S.-R.); 2 Departamento de Ciências Farmacêuticas, Universidade Federal de Pernambuco-UFPE, Recife-PE 50670-901, Brazil; magda.ferreira00@gmail.com (M.R.A.F.); phtech@uol.com.br (L.A.L.S.); 3 Departamento de Análises Clínicas, Universidade Estadual de Maringá-UEM, Avenida Colombo, 5790, Maringá-PR 87020-900, Brazil; terezinha.svidzinski@gmail.com; 4 Departamento de Infectologia, Universidade Federal do Rio Grande do Norte-UFRN, Rua Conego Monte, 110, Quintas, Natal-RN 59084-100, Brazil; evepipolo@gmail.com; 5 Laboratório de Micologia Clínica, Departamento de Análises Clínicas, Universidade Estadual Paulista-UNESP, Araraquara-SP 14801-902, Brazil; rehepi@gmail.com (R.H.P.); ana.marisa@uol.com.br (A.M.F.A.); giannini@fcfar.unesp.br (M.J.S.M.-G.)

**Keywords:** *Candida* spp., oral candidiasis, kidney transplant recipients, virulence factors, *Eugenia uniflora*

## Abstract

This study evaluated the influence of the extract of *Eugenia uniflora* in adhesion to human buccal epithelial cells (HBEC) biofilm formation and cell surface hydrophobicity (CSH) of *Candida* spp. isolated from the oral cavity of kidney transplant patients. To evaluate virulence attributes in vitro, nine yeasts were grown in the presence and absence of 1000 μg/mL of the extract. Adhesion was quantified using the number of *Candida* cells adhered to 150 HBEC determined by optical microscope. Biofilm formation was evaluated using two methodologies: XTT (2,3-bis(2-methoxy-4-nitro-5-sulfophenyl)-2*H*-tetrazolium-5-carboxanilide) and crystal violet assay, and further analyzed by electronic scan microscopy. CSH was quantified with the microbial adhesion to hydrocarbons test. We could detect that the extract of *E. uniflora* was able to reduce adhesion to HBEC and CSH for both *Candida albicans* and non-*Candida albicans* *Candida* species. We also observed a statistically significant reduced ability to form biofilms in biofilm-producing strains using both methods of quantification. However, two highly biofilm-producing strains of *Candida tropicalis* had a very large reduction in biofilm formation. This study reinforces the idea that besides growth inhibition, *E. uniflora* may interfere with the expression of some virulence factors of *Candida* spp. and may be possibly applied in the future as a novel antifungal agent.

## 1. Introduction

*Candida* species are micro-organisms of medical interest due to the high frequency with which they colonize and infect the human host. *Candida* spp. figure as colonizing yeasts of the oral mucosa in about 20–80% of healthy adults without evidence of infection [1]. Nevertheless, depending on the immune status of the host and virulence attributes of *Candida* spp., these micro-organisms may become pathogenic, specifically within particular groups of patients, such as transplant recipients, AIDS patients, and those under treatment with broad-spectrum antibiotics [2,3].

Oral candidiasis is a common opportunistic infection in kidney transplant recipients maintained under immunosuppressive therapy. Several studies have shown that the oral cavity of these individuals is a favorable microenvironment for the development of infections [4,5]. It is also important to emphasize that a study performed by our group revealed a statistically significant relationship between oral candidiasis and the use of dental prosthesis in renal transplant patients [2].

*Candida albicans* is still the most virulent and frequently isolated species of the *Candida* genus from both superficial and systemic infections [6]. However, recent studies have reported an increased frequency of candidiasis caused by non-*C. albicans Candida* (NCAC) species, each with its own specific peculiarities and natural history [6,7,8]. 

Several *Candida* species have been isolated from the oral cavity of the human host. Despite the fact that *C. albicans* is still the main etiological agent of oral candidiasis, other species such as *Candida tropicalis*, *Candida dubliniensis*, *Candida krusei*, *Candida glabrata*, the *Candida parapsilosis* species complex, and other yeasts have also been frequently isolated from this body site [8,9]. 

The transition from commensal organisms to pathogens is attributable to an extensive repertoire of virulence factors selectively expressed under suitable predisposing conditions [10,11]. Among *Candida* spp., the main putative virulence factors include the ability to adhere to human buccal epithelial cells (HBECs), yeast-to-hyphae transition (morphogenesis), phenotypic switching, secretion of hydrolytic enzymes, and biofilm formation [12].

Adhesion is the first step for the development of candidiasis and may occur in either host cells or on medical device surfaces, often leading to biofilm formation. Thus, adhesion is a key step for an infectious process, which is influenced by the composition of yeast cell walls as well as the characteristics of the surface to which yeast cells adhere [7,13]. 

Biofilms are communities of micro-organisms attached to either biotic or abiotic surfaces, embedded in a matrix of extracellular polymeric substance difficult to eliminate. Biofilm formation is a potent virulence factor of *Candida* spp., as it confers significant tolerance to antifungal therapy, mainly by limiting the penetration of substances through the extracellular matrix [14] and resistance to phagocytic cell attacks [15,16]. Perhaps the most clinically relevant biofilm-specific property is the development of antifungal resistance of cells composing it, where minimal inhibitory concentrations (MICs) can be up to 1000-fold higher than those found for planktonic cells [16,17]. 

Considering that resistance in some *Candida* species against antifungal drugs currently used to treat oral candidiasis may occur, in conjunction with the growing number of immunocompromised patients, including kidney transplant recipients, alternative therapeutic sources must be sought to treat this infirmity [6,8,18].

Brazil emerges as an important botanical material supplier for the international pharmaceutical market due to its rich biodiversity in plants with pharmacological activities. Therefore, there is a field with potential relevance for the search of new antifungal compounds of plant origin [19].

Herbal medicine is an important component of complementary and alternative medicine, and its importance is now recognized worldwide [20]. *Eugenia uniflora* is a native plant of South America, belonging to the Myrtaceae family, which includes species that contain phenolic compounds as the predominant constituents, popularly known in Brazil as “pitangueira”. It is an aromatic species and its essential oil has pharmacological properties, which are well characterized in the literature as antioxidants and antimicrobial agents [21,22]. Currently, its anti-*Candida* activity has been described [23,24,25].

Besides inhibition of growth, previous works performed by our group demonstrated the direct interference of *E. uniflora* components with *Candida albicans* expression of virulence factors in vitro and in vivo [26,27]. We found that this natural product greatly reduced hypha formation after morphogenesis induction in serum, Spider medium, and N-Acetyl d-Glucosamine (GlcNac) and impaired the ability to secrete phospholipase and proteinase. Oral candidiasis was attenuated in a murine model of infection and several proteins mainly related to cellular structure were differentially expressed in the presence of this natural product, as determined by proteomics analysis [27]. 

Considering our previous promising findings, we performed in vitro tests to examine the influence of the extract of the leaves of *E. uniflora* on the expression of some other important *Candida* spp. virulence factors in vitro, directly involved in oral candidiasis, including adhesion to HBECs, biofilm formation and cell surface hydrophobicity (CSH) of clinical isolates obtained from the oral cavity of kidney transplant patients in Brazil. This study will contribute for an increased knowledge upon the direct interaction of natural products with antifungal properties with the expression of virulence factors in vitro of a reasonable number of *Candida* different species.

## 2. Results and Discussion

We selected 42 strains of *Candida* spp. belonging to our mycological culture collection, previously isolated from the oral cavity of kidney transplant patients, in order to evaluate a reasonable number of *Candida* isolates belonging to different species [2]. They have been chosen as follows: 26 strains were *C. albicans* (61.9%), while 16 isolates (38.1%) corresponded to NCAC species, such as *C. tropicalis* (4 strains; 9.5%), *C. parapsilosis* (3 strains; 7.1%), *C. glabrata* (3 strains; 7.1%), *C. orthopsilosis* (2 strains; 4.8%), *C. metapsilosis* (2 strains; 4.8%), and *C. dubliniensis* (2 strains; 4.8%). 

This study aimed to evaluate the influence of the *E. uniflora* extract on the expression of some virulence factors in vitro of *Candida* species. In a previous study, Ferreira et al. [23] performed a screening of antifungal activities of medicinal plants from Northeast Brazil, including 30 different vegetal crude extracts and revealed *E. uniflora* as the most active natural product against *Candida* spp. Therefore, we selected the extract of this plant to perform the present study. The minimum inhibitory concentration (MIC) of *E. uniflora* extract was 312.5 µg/mL for *C. albicans*, *C. dublinienis* and *C. parapsilosis* complex species, while *C. tropicalis* and *C. glabrata* needed a slightly higher concentration (MIC equal to 625 µg/mL). Therefore, we decided to use a concentration higher than the MIC to perform the experiments, since we found that *Candida* cells are damaged but not completely unviable at this concentration. For this purpose, a concentration of 1000 μg/mL of the referred natural product was used to perform the in vitro virulence attributes tests in the presence of the extract, a lower concentration than the one used in our group previous publication, because it showed the same effect [26].

**Figure 1:**
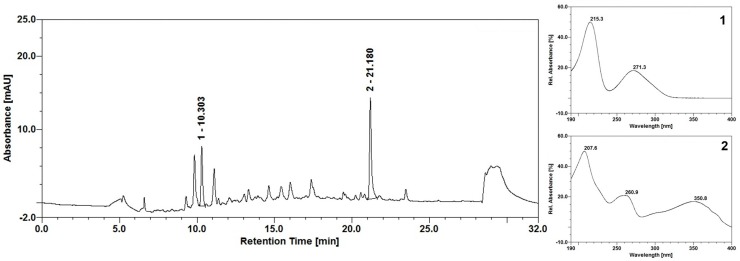
Chromatographic profile of *Eugenia uniflora* extract and UV spectra corresponding to peaks (**1**) gallic acid and (**2**) myricitrin.

The chromatographic fingerprint of *E. uniflora* extract is shown in Figure 1. In the chromatographic profiling obtained with the *E. uniflora* extract, a peak correspondent to gallic acid was identified, eluting at a retention time (Rt) of 10.3 min; the presence of the majoritarian compound, the derivative flavonoidic myricitrin, eluted with Rt = 21.18 min. The contents of gallic acid and myricitrin were calculated based with the calibration curves of the respective standards, and the results found were 0.16 g% ± 0.0017 (1.42) and 0.43 g% ± 0.0006 (0.01), respectively. Much research has shown the anti-*Candida* activity potential of plant-derived compounds such as flavonoids and essential oils [28,29,30]. 

The antifungal action exerted by plant-derived compounds is related to the amount of the compound present in the extract under analysis [31]. The majoritarian compounds isolated from the *E. uniflora* extract used in our study presented gallic acid and myricitrin, and in this context several studies have demonstrated the anti-*Candida* action of these compounds with an MIC range similar to our results [31,32,33,34,35]. 

**Figure 2:**
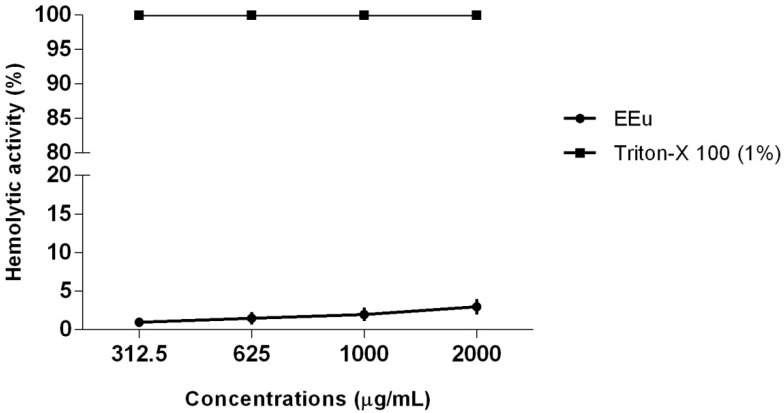
Hemolytic activity of *Eugenia uniflora* extract to human erythrocytes. Human erythrocytes (5 × 10^7^ cells/mL) were incubated for 1 h at 37 °C with different extract concentrations. Triton-X 100 (1%) and washing buffer were used as positive and negative controls, respectively. The hemoglobin released from lysed erythrocytes was measured at 540 nm on a UV-Vis Spectrophotometer. The absorbance values for each sample were subtracted from the negative control and the hemolytic activity (%) was calculated. The experiment was performed in triplicate.

Considering the possible adverse effects associated with the use of antifungal agents, of which the action may target eukaryotic fungal cells, it is necessary to analyze the toxicity of natural products with possible antifungal activity [6,36]. Therefore, we performed a cytotoxicity assay of *E. uniflora* extract against human erythrocytes and HBEC. The possible hemolytic activity of the extract was determined by measuring the lysis of human red blood cells suspension in a spectrophotometric assay. In this experiment, Triton X-100 1% (*v*/*v*) was used as a positive control and induced total erythrocytes lysis. We could observe that the *E. uniflora* extract showed no significant effect on erythrocytes lysis (Figure 2), even with the double of the concentration used to perform all the experiments of the present study (2000 μg/mL; Figure 2).

**Figure 3:**
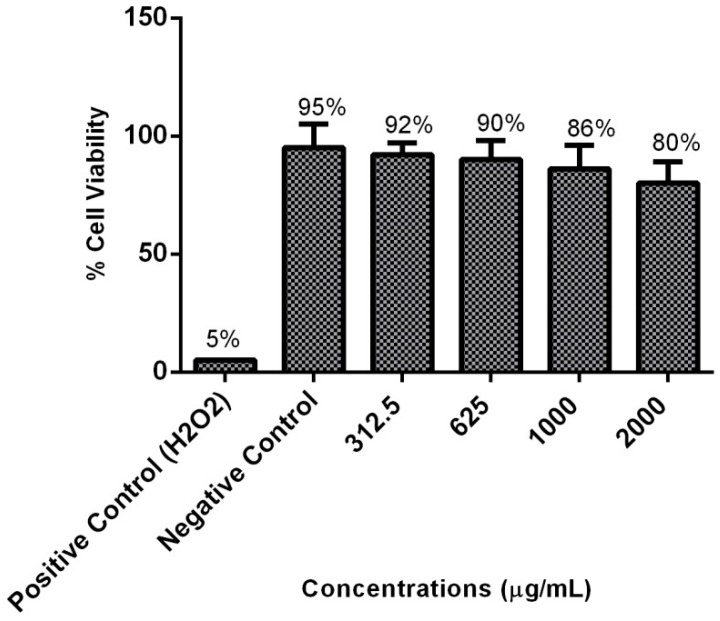
Cytotoxicity assay of *E. uniflora* extract to human buccal epithelial cells (HBECs). Cells were exposed for 1 h at 37 °C to different concentrations of extract, stained with trypan blue, and counted under light microscopy (10×). For each replicate, 150 epithelial cells were counted. The assay was performed in triplicate.

Concerning cytotoxicity to HBEC, the assay was performed by placing the epithelial cells in contact with different concentrations of the extract and subsequently stained with trypan blue dye. Of note, some of the epithelial cells not incubated in the presence of the extract were also unviable. This finding is expected, because they were collect from a human volunteer by scrapping. The results showed that the majority of cells remained viable even after the exposure to different concentrations of the *E. uniflora* extract (Figure 3). These results, in combination with our previous study using A549 cell line [27] demonstrated that the *E. uniflora* extract showed to be a safe and nontoxic natural product at the concentrations tested.

**Figure 4:**
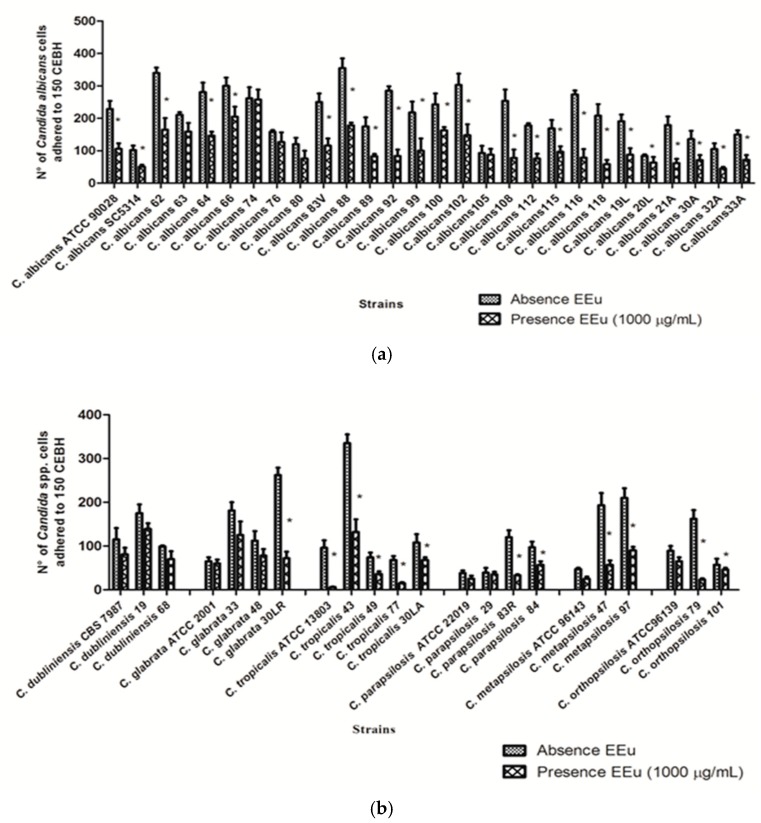
Evaluation of adhesion of *Candida* spp. after cells growth in the presence of 1000 µg/mL of *Eugenia uniflora* extract. (**a**) Reduction in adhesion ability of *Candida albicans* strains; (**b**) reduction in adhesion ability of non-*C. albicans Candida* (NCAC) strains. Each bar represents mean ± SD of the value of adhesion of all the strains of each species. Experiments were performed in triplicate. * represents a statistically significant difference between the control and test experiment to each strain (Mann-Whitney test, *p* < 0.05).

We further investigated adhesion to HBEC by the different *Candida* spp. isolates by determining the number of blastoconidia of each strain adhered to 150 HBEC observed with optical microscopy. *C. albicans* and NCAC isolates were able to adhere even when previously grown in the presence of the *E. uniflora* extract. *C. albicans* was proven to be the most adherent species (mean *C. albicans* adherence 209 ± 75 vs. mean NCAC adherence 125 ± 76, *p* < 0.05). Nevertheless, we observed that adhesion of yeast cells to HBEC was strongly reduced when cells were previously incubated with the *E. uniflora* extract. Comparing the results of the adhesion ability of isolates of *Candida* spp. grown in the presence of *E. uniflora* extract with the control group (no treatment), a statistically significant reduction in adhesion for most of the isolates was observed (Figure 4). Nevertheless, a large variation for all the strains regarding to the reduction of adhesion in the presence of the extract was observed when all the isolates were evaluated.

**Figure 5:**
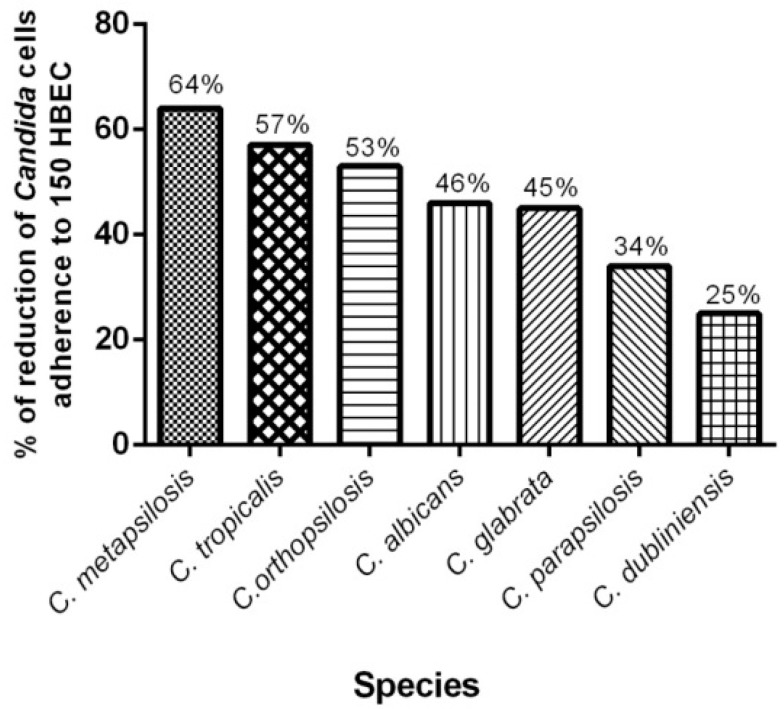
Percentage of reduction in adhesion ability of *Candida* species to HBEC after 1 h of incubation at 37 °C, 200 rpm. The experiment was performed in triplicate in the presence of 1000 µg/mL of *Eugenia uniflora* extract.

When we evaluated the percentage of reduction for each *Candida* species group separately, *C. metapsilosis* presented the greatest reduction, followed by *C. tropicalis*, *C. orthopsilosis*, *C. albicans*, *C. glabrata*, *C. parapsilosis* and *C. dubliniensis*. We also highlight strain 79 of *C. orthopsilosis*, which was the clinical isolate with highest percentage of reduction (Figure 5). Nevertheless, a limitation in our study is the number of representative strains of each species. We have used strains obtained from a kidney transplant recipients cohort previous investigation from our group and the prevalence of *C. albicans* was higher than the other species, as described everywhere [37,38]. Therefore, for this reason, the number of NCAC strains is lower and definitely may have influenced our results.

A comparison of the average values of the number of *Candida* cells adhered to 150 HBECs revealed a statistically significant reduction in adhesion for both *C. albicans* and NCAC species when evaluated separately, when previously grown in the presence of the extract (Table 1).

To the best of our knowledge, there are no other publications reporting an impairment of adhesion of *Candida* spp. to HBEC due to the extract of *E. uniflora*. Our results showed a trend of a remarkable reduction in adhesion of *Candida* species when grown in the presence of this natural product, reinforcing that, besides growth inhibition, this extract might directly interfere on *Candida* spp. adhesion to buccal epithelia. Therefore, it could be particularly useful for prevention of oral candidiasis, because adhesion is also needed for colonization in the oral cavity, which is always considered a previous step to infection [39]. 

A few other authors have reported antiadhesion effects of other natural products in *Candida* species. Thaweboon et al. [40] evaluated the effect of *Phyllanthus emblica* Linn. on *Candida* adhesion to oral epithelium and denture acrylic. They have found that the ethanolic extract obtained from the fresh fruits of *Phyllanthus emblica* Linn. was able to reduce adhesion of clinical isolates of *C. albicans* to buccal epithelial cells.

De Paula et al. [41] showed that eugenol, which is the main active phenylpropanoid component of the essential oil from many aromatic plants, impaired the ability of *C. albicans* to adhere to Hep2 cells and polystyrene surface. Lopes et al. [42] also demonstrated that phlorotannins from the brown seaweed *Fucus spiralis* inhibited morphogenesis in *C. albicans* with the formation of pseudohyphae and limited ability to adhere to epithelial cells. Recently, Matsuura et al. [43] demonstrated that the extract of *Paulinia cupana* (guaraná) was able to reduce adhesion of *C. albicans* to HBEC, even despite the fact it did not reduce fungal growth, emphasizing the direct interaction of a natural product in adhesion, suggesting its potential use to prevent oral candidiasis. 

**Figure 6:**
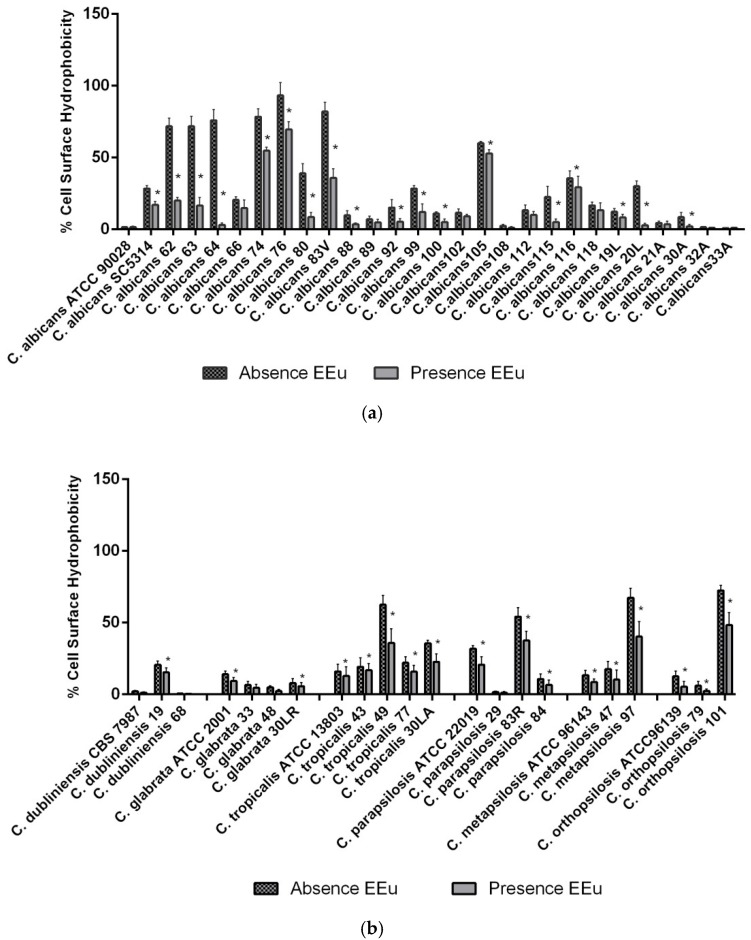
Evaluation of cell surface hydrophobicity (CSH) of *Candida* spp. after cell growth in the presence of 1000 µg/mL of *Eugenia uniflora* extract. (**a**) CSH reduction of *Candida albicans* strains. (**b**) CSH reduction of NCAC strains. Each bar represents mean ± SD of the value of adhesion of all the strains of each species. Experiments were performed in triplicate. * represents a statistically significant difference between the control and test experiment to each strain (Mann-Whitney test, *p* < 0.05).

CSH properties of oral *Candida* strains of the present study are shown in Figure 6 and Table 2. Our hydrophobicity assay showed that *E. uniflora* extract induced a statistically significant change in CSH levels of all *Candida* species tested (Figure 6). Most *Candida* species showed variable levels of affinity to the hydrocarbon phase. However, *C. albicans* strains presented a discrete higher affinity for the hydrocarbon phase than the other NCAC species. This finding may be influenced by the number of strains analyzed in each group. 

CSH is an intrinsic property of the external cell wall layer which makes *Candida* spp. cells close to each other, besides inducing aggregation. This force keeps optimal distance between adhesion molecules and host receptors, which lead to strong binding and finally irreversible adherence to mucosal membrane or other substrates. Adherence is an initial and important step for biofilm formation. Therefore, hydrophobicity might be a contributing factor to biofilm formation [44]. In addition, due to the fact that CSH of *Candida* spp. can affect cellular behavior as well as adhesion capacity, the reduction of the hydrophobic properties can lead to limitation in yeast colonization [45].

There are no published reports upon the action of the extract of *E. uniflora* on CSH of *Candida* spp. However, some studies evaluating the action of natural products in CSH have been performed. For instance, Zorić et al. [46] evaluated the action of oleuropein in the expression of *C. albicans* virulence factors. The authors showed that this compound interfered with CSH in *C. albicans*. Shirley et al. [47] determined in vitro effectiveness of *Plantago major* extract on the inhibition of *C. albicans* growth, biofilm formation, and CSH, which decreased at the highest concentrations tested.

Others studies have showed a positive effect on *Candida* spp. CSH, adhesion to epithelial cells and biofilm formation for a variety of naturally derived products or their constituents: eugenol [41], magnolol, and honokiol [48], the extracts of *Brucea javanica* and *Piper betle* [49]. 

**Figure 7:**
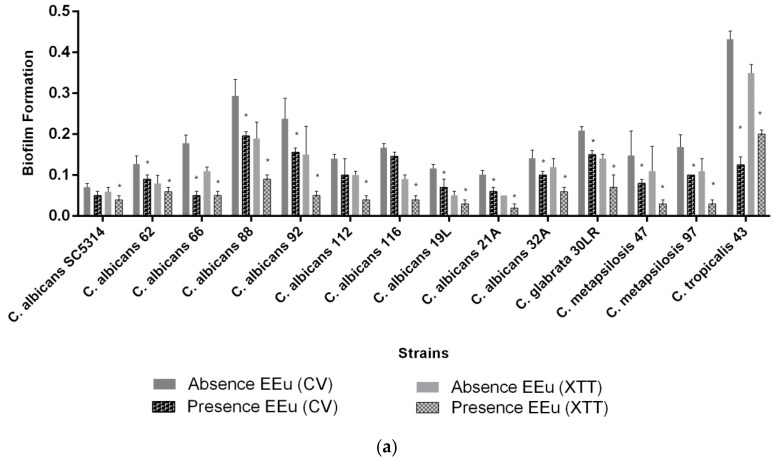

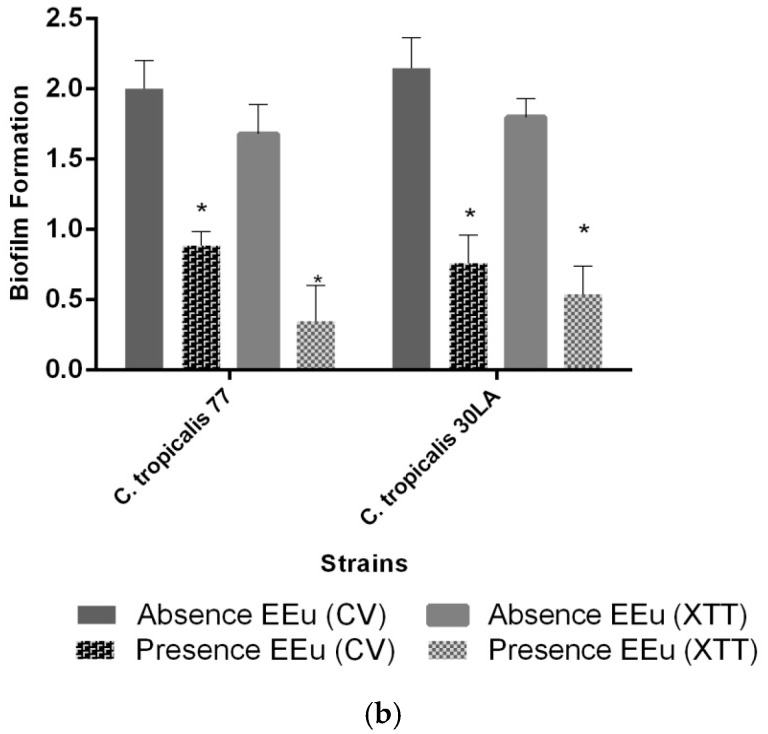
Reduction of biofilm formation in the presence of *E. uniflora* extract after incubation in YNB medium during 66 h, 37 °C, 75 rpm by crystal violet and XTT assay. (**a**) Reduction in biofilm formation of *Candida* spp.; (**b**) reduction in biofilm formation of *Candida tropicalis* strains highly biofilm formers. The experiment was performed in the presence of 1000 µg/mL of *Eugenia uniflora* extract. Each bar represents mean ± SD of the value of adhesion of all the strains of each species. * *p* < 0.05.

Regarding to the investigation of biofilm formation, according to the Stepanovick et al. [50] classification, we found that 38% (16 strains) of all isolates analyzed were able to form biofilm. Among the clinical isolates of *C. albicans*, 62% (10 strains) were able to form biofilm, while 38% (6 strains) of the NCAC isolates belonging to *C. glabrata*, *C. tropicalis* and *C. parapsilosis* complex species could also express this important virulence factor in vitro (Figure 7). Therefore, we only chose biofilm-forming strains to evaluate the effect of *E. uniflora* on this attribute of virulence of *Candida* spp.

Concerning to the effect of the *E. uniflora* extract on biofilm formation, *Candida* biofilms were quantified with two different methodologies: CV assay, which measures the total biomass of the biofilm, and the XTT assay, which measures biofilm metabolic activity. Of note, we found a positive correlation between the two methodologies used to quantify biofilm formation (*r* = 0.9698). We also observed a statistically significant reduced ability to fully express this virulence factor in vitro for all strains. The percentage of reduction ranged from 7 to 79% in isolates of *C. albicans* and from 3 to 95% in NCAC isolates (Figure 7a,b). This finding is particularly remarkable in our two *C. tropicalis* strong biofilm producers, which showed a notorious reduction in biofilm formation observed by the two methodologies tested in the presence of *E. uniflora* (Figure 7b). 

There are actually a few published reports upon the action of *E. uniflora* on micro-organisms’ biofilm formation. For instance, Oliveira et al. [51] showed that the hydroalcoholic extract of the fruit and infused leaves of this natural product has antibacterial activity against biofilm-forming bacteria. More recently, Zeuko’o et al. [52] analyzing the anti-*Candida* biofilm properties of Cameroonian plant extracts, observed that *E. uniflora* extract reduced biofilm formation of two clinical strains (*C. albicans* and *C. glabrata*) obtained from patients with vulvovaginal candidiasis. In fact, the number of studies investigating anti-biofilm properties of natural products in *Candida* spp. has steadily increased recently [47,53,54].

Most of the isolates that showed a significant reduction in biofilm formation belong to *C. albicans* species. However, some isolates of *C. glabrata*, *C. tropicalis*, and *C. metapsilosis* also showed impaired biofilm formation in the presence of *E. uniflora* extract. Considering that, even for commercially available antifungal drugs, each strain should be tested for MIC determination and that most of the anti-biofilm natural products published in the literature were tested in a very low number of strains, this finding cannot be neglected. Interestingly, we found a remarkable reduction of biofilm formation for the two strong biofilm producer strains of *C. tropicalis* proving that the vegetal extract may somehow interfere with this specific virulence factor. This finding is of great importance because, besides the fact that adhesion to buccal epithelia and CSH were reduced in the presence of the extract, biofilm formation is a potent virulence factor of *Candida* species, which confers resistance to antifungal therapies, limiting the penetration of substances through an extracellular matrix [7,55]. 

**Figure 8:**
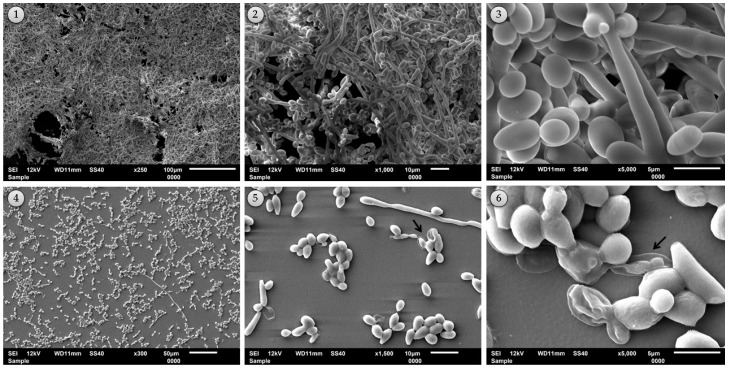
Scanning electron microscopy (SEM) images of the effect of *E. uniflora* extract on *Candida tropicalis* strain 77, a high biofilm former. SEM photomicrographs (250–300×, 1000–1500×, and 5000×) before (boards 1–3) and after (boards 4–6) treatment with 1000 µg/mL of *E. uniflora* extract. Black arrows indicate damage to *Candida* sessile cells in the presence of the natural product.

The property of reduction of biofilm formation by *E. uniflora* extract on a strong biofilm forming *C. tropicalis* isolate (strain 77) was analyzed by scanning electron microscopy (SEM). We could confirm that, besides reducing the amount of blastoconidia and hyphae, there is also clear damage generated in the cellular structure in the presence of the extract (Figure 8). We can notice the presence of roughness and a coarse surface in the cell being indicative of a supposed action on the cell wall.

If we consider that we have tested a higher number of strains than several other studies, this study showed that the extract of leaves of *E. uniflora* acted in an efficient way in reducing adhesion to HBEC, CSH and reducing biofilm formation. This is an interesting finding given that the process of *Candida* adhesion is the first step to biofilm formation, and the CSH is a property that exerts influence on adhesion [7]. Once again, it is important to emphasize that our highly biofilm-producing strains of *C. tropicalis* (strains 77 and 30LA) had extremely reduced biofilm formation in the presence of *E. uniflora* extract.

**Figure 9:**
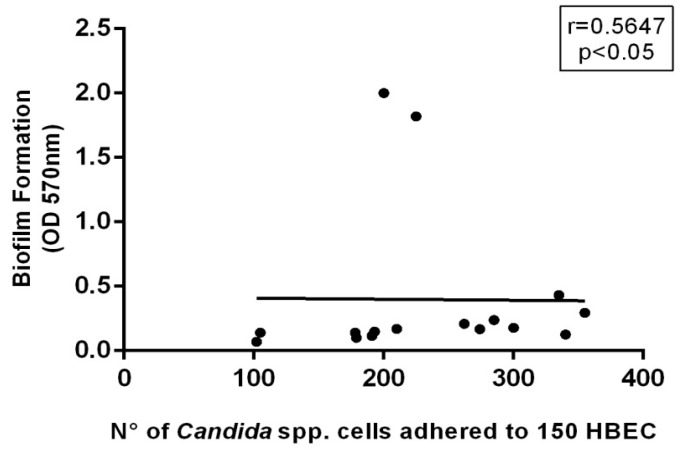
Correlation of *Candida* spp. adherence ability to HBEC and biofilm formation at polystyrene surface. Spearman coefficient was used to assess the correlation.

We next evaluated a possible correlation between the virulence factors determined in vitro using the Spearman coefficient. The results showed a moderate correlation between the adhesion capacity of HBEC and biofilm formation (crystal violet stained; Figure 9), meaning that there is a trend that the most adherent strains to the buccal epithelia are also moderate to strong biofilm producers, with remarkable production of exopolymeric matrix. Therefore, these results emphasize that *E. uniflora* may reduce the expression of these important virulence factors in *Candida* spp., specifically because adhesion is the first step for biofilm formation. 

Contradictory to these findings, we observed that there was no statistically significant correlation between CSH and the other virulence factors analyzed.

CSH is a factor related with fungal cell walls and is usually considered a good indicator of adhesion ability. Despite some earlier studies have indicated a positive correlation between CSH and adhesion to polystyrene, acrylic, and tissue surfaces, a few others suggested there is no correlation between adhesion to different substrates and CSH, meaning that adhesion of *Candida* to either biotic or abiotic surfaces is a complex mechanism [44,56,57,58].

*Candida* cells with reduced CSH may also present impaired biofilm formation in some circumstances. However, this cannot be recognized as a general rule because meaningfully reduced ability of biofilm formation has also been observed for yeasts that did not show any changes in their hydrophobic properties [43,45].

The extract of *E. uniflora* used in our study showed in its chemical characterization considerable amounts of phenolic compounds, such as hydrolysable tannins and flavonoids (unpublished data). Similar to azoles, tannins derived from vegetable target lipids of host cell membranes [59]. Moreover, phenolic compounds impair growth and biofilm formation in *C. albicans,* possibly through the suppression of genes responsible for adhesion and morphogenesis [60]. The presence of phenolic compounds in the extract of *E. uniflora* may have led to a reduced ability to adhere to HBECs, CSH, and biofilm formation in some of the strains tested. 

Taking the results from our previous publication, specifically the impairment of hypha formation and decreased recognition from phagocytic cells [27] and the findings of the present study, we hypothesize that the extract of *E. uniflora* interacts directly with the cell wall of *Candida* spp., because several adhesins, such as Hwp1, directly involved with biofilm formation [61] and adhesion [39] are present at this cellular component. When *Candida* cells were grown in the presence of the extract, accumulation of components of the natural product on the cell wall may have led to decreased adhesiveness, an important step also related to biofilm formation and CSH. Decreased cell fitness may have influenced the secretion of exopolymeric matrix, as observed with our biofilm formed on polystyrene surfaces, quantified with crystal violet staining and analyzed by SEM. We are currently working on the mechanisms of action of *E. uniflora*, including the investigation of the influence of cell wall perturbators (such as Congo Red, Calcofluor White, and Caffeine) and ergosterol quantification to confirm our hypothesis. 

## 3. Materials and Methods

### 3.1. Plant Material and Eugenia uniflora Extract

The leaves of *E. uniflora* were collected in July 2009 at the city of Ceara-Mirim, in the northeast region of the State of Rio Grande do Norte, Brazil. Of note, we chose this time of the year for plant collection, because our rainy season (when the temperature is also lower in the South Hemisphere) is ideal for flowering, fruiting and fitness of *E. uniflora*. The plant was identified at the Herbarium of the Federal University of Rio Grande do Norte (Department of Botany, Zoology and Ecology, Biosciences Center) and a voucher specimen was deposited (No. 11763). The leaves of *E. uniflora* were dried at room temperature and ground into powder. The crude extract was obtained using 10 g of the powder with 100 mL of acetone: water (7:3, *v*/*v*) as a solvent by turbo extraction. The resulting extract was filtered and concentrated under reduced pressure (RV10 Basic, IKA^®^, Campinas, Sao Paulo, Brazil) at 40 °C, 150 rpm. The concentrated extract was lyophilized to –64 °C, 0.006 mBar (Model 101, Liotop^®^, Sao Carlos, São Paulo, Brazil), and the extract was stored at 4 °C. 

### 3.2. High-Performance Liquid Chromatography (HPLC) Analysis of Eugenia uniflora Extract

The extract of *E. uniflora* (acetone: water, 7:3, *v*/*v*) was analyzed by HPLC through an Ultimate 3000 system (ThermoScientific^®^, Waltham, Massachusetts, MA, USA). The chromatographic separation was performed using a C_18_ column (Dionex^®^). Chromeleon 6.8 (Dionex^®^, Sunnyvale, California, CA, USA) software was used for data acquisition and processing. The next steps were performed according to De Araújo et al [62]. The peaks of substances were identified through the retention times and UV spectra. Subsequently, the peaks were confirmed by spiking the sample with the standards. 

### 3.3. Strain and Culture Conditions

We evaluated a total of 42 *Candida* spp. clinical isolates obtained from the oral cavity of kidney transplant recipients, belonging to the Medical and Molecular Mycology Laboratory, Clinical and Toxicological Analyses Department, at the Federal University of Rio Grande do Norte. The isolates were stored at –80 °C in YPD containing 20% glycerol. Cells were defrosted on ice and 100 µL of each strain was added to 5 ml of YPD liquid medium (dextrose 20 g/L, peptone 20 g/L, yeast extract 10 g/L) and incubated in a rotator shaker (TE-420, Tecnal^®^ Piracicaba, Brazil) at 35 °C, 200 rpm, for 48 h for reactivation and verification of cell viability. Subsequently, 100 µL of cell suspensions was inoculated on the surface of Sabouraud Dextrose Agar (SDA; Oxoid, Basingstoke, Hampshire, UK) using a Drigalsky loop. The strains were identified phenotypically and molecularly as described in Chaves et al. [2]. 

### 3.4. MIC of Eugenia uniflora Extract 

MIC was based on Clinical protocol M27-A2 and Laboratory Standards Institute (CLSI) (Wayne, PA, USA) with adaptations for natural products [63]. The inoculum of all strains tested was obtained from 48 h cultivation in SDA at 35 °C and an initial suspension prepared according to McFarland scale 0.5 standard (1 a 5 × 10^6^ cells). Then, two serial dilutions were made, the first in saline solution (1:50) and the second in Mueller–Hinton Growth medium (Difco) (1:30). After that, aliquots of 100 μL of the final inoculum solution were dispensed in microtiter plates of 96 wells containing 100 μL of various concentrations of the *E. uniflora* extract. The range tested was 10,000 µg/mL to 19.53 µg/mL. Finally, the plates were incubated at 37 °C and test reading taken after 48 h incubation. MIC was considered the lowest concentration of the *E. uniflora* extract capable of inhibiting 50% of the growth of each strain, taking as reference the respective positive control (treated in the same manner, but without the extract added to yeast cells). 

### 3.5. Hemolytic Activity of Eugenia uniflora Extract on Human Erythrocytes

Hemolytic activity was performed according to Silva-Filho et al. [64] with some modifications. A healthy adult donor provided blood for the in vitro experiments. 5 mL of venous blood was collected and placed in EDTA tubes (ethylene-diamine-tetraacetate, Labtest, Lagoa Santa, Brazil) and promptly centrifuged (refrigerated centrifuge, ALC, Model PK121R, Milan, Italy) at 1100× *g* for 10 min at 4 °C. Plasma was carefully aspirated and the buffy coat was removed. The pellet containing erythrocytes was washed three times in phosphate-buffered saline (pH = 7, PBS) and suspended in PBS to a final suspension of 5 × 10^7^ cells/mL counted with a hemocytometer. Two mL of cell suspension was incubated for 1 hour at 37 °C with different *E. uniflora* extract concentrations (312.5, 625, 1000, and 2000 µg/mL). Triton-X 100 (1%) and washing buffer were used as positive and negative controls, respectively. The amount of hemoglobin released from lysed erythrocytes was measured at 540 nm on a UV-Vis Spectrophotometer (Biochrom Libra S32, Cambrigde, UK). The absorbance values for each sample were subtracted from the negative control and the percentage of hemolytic activity (%) was calculated. The hemolytic activity (%) = (A − A_0_)/A_x_ − A_0_) × 100, where A is the O.D. of tube test, A_0_ is the O.D. of negative control, and A_x_ is the O.D. of Triton-X 100 (1%). The experiment was performed in triplicate.

### 3.6. Cytotoxicity Assay of E. uniflora Extract to HBEC

The cytotoxicity assay of *E. uniflora* extract to HBEC was performed according to Crowley et al. [65] with some modifications. HBECs were collected as described in Section 3.7.1. A suspension with 5.0 × 10^5^ cells/mL was prepared and treated with different *E. uniflora* extract concentrations (312.5, 625, 1000, and 2000 µg/mL) during 1 h, at 37 °C, while positive control (viable cells) epithelial cells were treated by the same manner, except by the fact that they were not incubated in the presence of the extract. Cells treated with hydrogen peroxide (Sigma-Aldrich, St. Louis, MO, USA) constituted the negative control (dead cells). Cells were washed three times with PBS (pH = 7.0) after incubation and 50 µL of each tube was mixed with an equal volume of trypan blue (Sigma-Aldrich) solution (0.4%). The number of viable cells was determined by counting 150 HBEC with the operator blinded to the nature of the material on the slide. The percentage of cells viability was calculated using the following equation: %Viability = (Number of colorless cells/Total number of cells) × 100. The experiment was performed in triplicate.

### 3.7. Candida spp. Virulence Factors

For *Candida* spp. virulence factors tested in vitro, *C. albicans* ATCC90028, *C. albicans* SC5314, *C. tropicalis* ATCC13803, *C. dubliniensis* CBS7987, *C. glabrata* ATCC2001, *C. parapsilosis* ATCC22019, *C. orthopsilosis* ATCC96139, and *C. metapsilosis* ATCC96143 were used as reference controls. 

#### 3.7.1. *Candida* spp. Adherence to HBEC in the Presence of *Eugenia uniflora*

*Candida* cells were grown overnight to the stationary phase in NGY liquid medium (0.1% Neopeptone (Difco), 0.4% glucose, and 0.1% yeast extract (Difco)) with 1000 μg/mL of concentration of *E. uniflora* extract at 30 °C. For adhesion assay experiments, HBECs were collected from a unique healthy volunteer (not colonized by yeasts). This procedure was performed in the day of the experiment by gently rubbing a sterile swab on the mucosal surface of the cheeks. Subsequently, the sterile swab was placed in sterile conic tubes containing 5 mL sterile saline at 4 °C until the moment of the experiment. The mixtures containing a ratio of 10 yeast cells per HBEC were incubated at 37 °C for 45 min with shaking (200 rpm) and then cells were vortexed, formalin-fixed, vortexed again to remove nonadherent cells, and transferred to a microscope slide. The number of *Candida* cells adhering to 150 HBEC was determined with the operator blinded to the nature of the material on the slide [66]. Tests were done in triplicate. The same conditions, except with the absence of the extract added in the NGY liquid medium, were followed for control experiments. Of note, CFU counts after 48 h incubation on SDA plates were performed with *Candida* spp. cells grown in NGY both in the absence and presence of the extract, to guarantee that test and control experiments contained the same number of viable cells. 

#### 3.7.2. CSH

*Candida* isolates were cultured in YNB medium (“Yeast Nitrogen Base”, Difco^TM^, Detroit, Michigan, MI, USA) with 50 mM of glucose (d-glucose monohidratada P.A., Cinética) for 18 h at 37 °C in a shaker (Tecnal, TE-420, SaoPaulo, Brazil) at 200 rpm, and absorbance was adjusted to OD_600nm_ = 1.0. For each strain, 2 mL of cell suspension was transferred to four glass tubes, representing one test group and one control group. An aliquot of 0.4 mL of xylene was added to each tube. The test and control tubes were incubated in a water bath (TE-056 Mag, Tecnal, São Paulo, Brazil) at 37 °C for 10 min, then shaken for 30 s, and returned to the water bath for another 30 min for a separation of the aqueous phase and of xylene. The aqueous phase was aspirated and transferred to a clean tube. The absorbance was measured at 520 nm. Hydrophobicity was given by the fraction [(Co − CH)/Co] × 100, where Co represents an OD_520nm_ of the control tube and CH represents OD_520nm_ of the test tube [44]. Each strain was tested in triplicate. For the test group cells, 1000 μg/mL of *E. uniflora* extract was added to the culture medium used to standardize the inoculum.

#### 3.7.3. *Candida* spp. Biofilm Formation in the Presence of *Eugenia uniflora*

Biofilms were performed and stained with crystal violet according to Chaves et al. [2]. For the test group cells, 1000 μg/mL of *E. uniflora* extract was added to the culture medium for inoculum standardization. For the XTT assay, the biofilm-coated wells of microtiter plates were washed twice with 150 µL of PBS and 100 mL of the XTT/menadione (Sigma-Aldrich) solution (1 µM of menadione) was added to each well containing a prewashed biofilm as well as to negative control wells. The plates were covered with aluminum foil and incubated in the dark for 2–3 h at 37 °C. Subsequently, 75–80 mL of the solution was removed (resulting colored supernatant from each well) and transferred into the wells of a new microtiter plate. The solution was measured with a microtiter plate reader (SpectraMAX 340 Tunable Microplate Reader; Molecular Devices LLC., San Jose, California, CA, USA) at 490 nm [67].

#### 3.7.4. Analysis of *Candida* spp. Biofilm Formation in the Presence of *Eugenia uniflora* Extract by Electronic Scan Microscopy (SEM)

The preparation of the samples for the SEM documentation was performed from pure cultures of the yeasts, grown in 20 mL of YPD medium contained in 50 mL conical tubes in an orbital shaker (TE-420, Tecnal^®^ Piracicaba, Brazil) (150–180 rpm) at 30 °C for 18–20 h. Subsequently, cells were washed in PBS for three times at 2500 rpm for 5 min. Thereafter, cells were initially suspended in PBS and adjusted to a concentration of 1 × 10^6^/mL in RPMI, by the McFarland scale 0.5 standard. Next, 300 μL of cell suspension was dispensed into the wells of a 12-well microtiter plate (TPP, Zollstrasse, Trasadingen, Switzerland) in which a 2-mm-thick glass disk was deposited. Plates were incubated a 37 °C for 24 h to allow biofilm formation on glass disks, without shaking. After the incubation period, disks were washed with sterile PBS and fresh medium was added (control experiment). For the evaluation of the action of *E. uniflora* on biofilm formation, fresh medium with 1000 μg/mL of the natural product was added to the wells (test experiments). Plates containing both control and test experiments were incubated for another 48 h at 37 °C. Plates were washed thrice with PBS and the next steps were equally performed for either treated or untreated biofilms (control experiments). To this end, 1 mL of 3% glutaraldehyde and 2% paraformaldehyde in 0.1 M potassium phosphate buffer, pH 7.4 were added to each well for sample fixation. Subsequently, plates were subjected to six washes with pure buffer solution at 15 min intervals and after were fixed in 1% osmium tetroxide for 16 h. After the incubation period, the material was again subjected to six washes with the same buffer solution and subjected to dehydration with increased concentrations of ethyl alcohol (30%, 50%, 70%, 80%, 90%, 95%, 100%). The samples were then dried in a Critical Point Dryer (CPD-030, Balzers, Oberkochen, Germany), and mounted for gold plating (DENTON VACUM metallizer, DESK II model, Freehold, NJ, USA), analyzed, and electromicrographed in a scanning electron microscope (JEOL, model JSM 5410, Chiyoda, Tokyo, Japan) operated at 15 KV [68].

### 3.8. Statistical Analysis

Data were analyzed using the statistical software “GraphPad, Prism” version 5.0, (GraphPad Software, La Jolla, California, CA, USA). Results were presented as mean ± standard deviation, and differences were analyzed by the Mann–Whitney test, while the Spearman coefficient was used to assess the correlation between the different techniques used to evaluate the expression of virulence factors in vitro. For all the analyses, P was considered a default value of 0.05 and the confidence interval of 95%.

## 4. Conclusions

In conclusion, this was the first study to evaluate the effects of *E. uniflora* extract on adhesion to human buccal epithelia, CSH, and biofilm formation, which are important virulence factors of *Candida* spp. Our results showed that the extract interferes with adherence of *Candida* spp. to HEBC and CSH and it is able to reduce biofilm formation for biofilm producing strains. Of note, *C. tropicalis* highly biofilm-producing strains showed a remarkable reduction in biofilm formation caused by the extract. Taken together, this finding and our previous publication, which also showed limited filamentation and hydrolytic enzymes expression for *C. albicans,* demonstrated a direct influence of the referred natural product with the expression of virulence attributes of *Candida* spp. in vitro. Therefore, it may be a viable alternative for the use to prevent oral candidiasis, specifically in denture wearers. Our group is currently working on the elucidation of the chemical compounds responsible for antifungal properties and the mechanism of action for both growth inhibition and interaction with *Candida* spp. virulence factors in vitro of *E. uniflora.*

## Figures and Tables

**Table 1:** Average of adhesion ability of *C. albicans* spp. isolates obtained from the oral cavity of kidney transplant recipients after 1h of incubation at 37 °C, 200 rpm. The experiment was performed in the presence of 1000 µg/mL of *Eugenia uniflora* extract (in triplicate).

	N° of Cells Adhered to 150 HBEC
	Absence of EUE ** vs. Presence of EUE **
All *Candida* spp. (N = 42 strains)	172 ± 86 vs. 87 ± 51 *
*Candida albicans* (N = 26 strains)	209 ± 75 vs. 108 ± 51 *
NCAC species (N = 16 strains)	125 ± 76 vs. 61 ± 39 *

* *p* < 0.05, ** EUE: *Eugenia uniflora* extract (1000 µg/mL).

**Table 2:** CSH values of *Candida* spp. strains. Average of CSH of *C. albicans* spp. isolates obtained from the oral cavity of kidney transplant recipients. The experiment was performed in the presence of 1000 µg/mL of *Eugenia uniflora* extract (in triplicate).

Species	N° of Strains	Mean + SD (Range)	
		CSH (% Value)	
		Absence of EUE **	Presence of EUE **
*C. albicans*	26	25.5 ± 0.9 (0.9 − 93.3)	16.9 ± 0.8 (1.0 − 69.5) *
Overall NCAC species	16	20.4 ± 1.9 (0.6 − 72.4)	12.6 ± 1.2 (1.2 − 48.4) *

* *p* < 0.05, ** *Eugenia uniflora* extract (1000 µg/mL).
